# Small noncoding RNAs and sperm nuclear basic proteins reflect the environmental impact on germ cells

**DOI:** 10.1186/s10020-023-00776-6

**Published:** 2024-01-20

**Authors:** Giulio Ferrero, Rosaria Festa, Laura Follia, Gennaro Lettieri, Sonia Tarallo, Tiziana Notari, Antonella Giarra, Carmela Marinaro, Barbara Pardini, Alessandra Marano, Giulia Piaggeschi, Carla Di Battista, Marco Trifuoggi, Marina Piscopo, Luigi Montano, Alessio Naccarati

**Affiliations:** 1 Department of Clinical and Biological Sciences, University of Turin, Regione Gonzole 10, 10043 Orbassano, Turin Italy; 2 Department of Computer Science, University of Turin, Corso Svizzera, 185, 10149 Turin, Italy; 3 Department of Biology, University of Naples Federico II, Via Cinthia, 21, 80126 Naples, Italy; 4 Italian Institute for Genomic Medicine (IIGM), c/o IRCCS Candiolo, SP 142 Km. 3,95, 10060 Candiolo, Turin Italy; 5 Check-Up PolyDiagnostic and Research Laboratory, Andrology Unit, Viale Andrea De Luca 5, 84131 Salerno, Italy; 6 Department of Chemical Sciences, University of Naples Federico II, Via Cinthia, 21, 80126 Naples, Italy; 7 Andrology Unit and Service of Lifestyle Medicine in UroAndrology, Local Health Authority (ASL) Salerno, Coordination Unit of the Network for Environmental and Reproductive Health (Eco-FoodFertility Project), S. Francesco di Assisi Hospital, 84020 Oliveto Citra, Salerno Italy; 8 PhD Program in Evolutionary Biology and Ecology, University of Rome Tor Vergata, 00133 Rome, Italy

**Keywords:** Environmental pollution, Male fertility, Small noncoding RNA, Sperm nuclear basic proteins, Transcriptomics

## Abstract

**Background:**

Molecular techniques can complement conventional spermiogram analyses to provide new information on the fertilizing potential of spermatozoa and to identify early alterations due to environmental pollution.

**Methods:**

Here, we present a multilevel molecular profiling by small RNA sequencing and sperm nuclear basic protein analysis of male germ cells from 33 healthy young subjects residing in low and high-polluted areas.

**Results:**

Although sperm motility and sperm concentration were comparable between samples from the two sites, those from the high-pollution area had a higher concentration of immature/immune cells, a lower protamine/histone ratio, a reduced ability of sperm nuclear basic proteins to protect DNA from oxidative damage, and an altered copper/zinc ratio in sperm. Sperm levels of 32 microRNAs involved in intraflagellar transport, oxidative stress response, and spermatogenesis were different between the two areas. In parallel, a decrease of Piwi-interacting RNA levels was observed in samples from the high-polluted area.

**Conclusions:**

This comprehensive analysis provides new insights into pollution-driven epigenetic alterations in sperm not detectable by spermiogram.

**Graphical Abstract:**

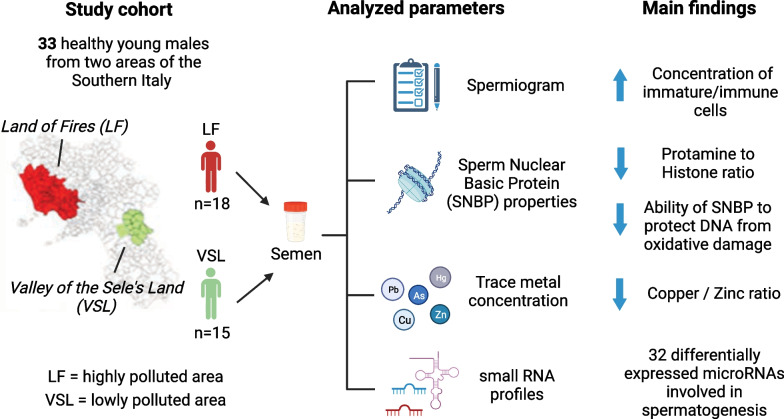

**Supplementary Information:**

The online version contains supplementary material available at 10.1186/s10020-023-00776-6.

## Background

Pollution has a marked impact on the reproductive health of living organisms with significant influences being shown on spermatozoa (Sharpe 2010; Lettieri et al. 2020; Montano et al. 2022), which are cells particularly sensitive to environmental changes (Bergamo et al. 2016; Bosco et al. 2018). Several pollutants are known to induce an imbalance of reactive oxygen species (ROS) (Piscopo et al. 2018; Montano et al. 2022): the resultant oxidative stress events have consequences on sperm morphology, number, and motility (Vecoli et al. 2016; Vecoli et al. 2017) and cause oxidative damage to sperm DNA (Lettieri et al. 2020). Spermatozoa appear to be particularly responsive to the pro-oxidant effects of environmental pollutants mainly for two factors: as a consequence of the small volume of the cytoplasmic space, resulting in reduced antioxidant defenses (Aitken et al. 2016), and also because sperm membrane lipids, particularly rich in polyunsaturated fatty acids, are the preferential target of ROS (Nowicka-Bauer and Nixon 2020). Therefore, at present, spermatozoa have been identified as an “ideal” biomarker of environmental pollution and an early warning sentinel for human health (Bergamo et al. 2016). Many biomonitoring programs are emerging to explore potential biomarkers suitable for evaluating the impact of pollution on human health, using semen, among other matrices (Longo et al. 2022). This is also in consideration that human infertility caused by environmental pollution is an increasing problem (Maric et al. 2021; Auger et al. 2022). Nowadays, semen health is mainly measured based on standard parameters; however, the combination of these traditional tools with advanced molecular approaches might allow a more detailed functional evaluation of sperm cells.

In mature human spermatozoa, two types of sperm nuclear basic proteins (SNBPs) pack the genome into the highly condensed sperm nucleus: protamines (PRM1 and PRM2) and histones (Rathke et al. 2014; Bao and Bedford 2016). In the human spermatozoa, the canonical ratios of protamines to histones (85%:15%) and of PRM1/PRM2 (0.8–1.2) are essential to ensure their fertilizing capacity (Oliva 2006). We have previously shown alterations in the properties of SNBPs in young men residing in polluted areas of Italy (Lettieri et al. 2020; Perrone et al. 2022). In the same individuals, we also observed altered macro and trace metal concentrations in their semen (Nunzio et al. 2022). Considering the relevant issue of the association between trace metals in semen and male infertility, still not fully explored (Lopez-Botella et al. 2021; Bhardwaj et al. 2021; Wirth and Mijal 2010), this can represent another biochemical link between environmental exposure and the observed molecular alterations. Moreover, data from the European project EXPOsOMICs (www.exposomicsproject.eu/) demonstrated a relationship between ultrathin particles or PM2.5 (fine particles) and omics profiles. Specifically, it has been shown that exposure to environmental pollutants change the expression profile of microRNAs (miRNAs): an altered expression of this class of small noncoding RNAs (sncRNAs) could be considered a marker of pollutant exposure or even an effect. miRNAs are pivotal post-transcriptional regulators of gene expression (Shang et al. 2023) and their role in spermatogenesis has been largely explored (Salas-Huetos et al. 2020). In the EXPOsOMICs project, it was observed that exposure to PM2.5 was associated with the altered expression of several circulating miRNAs (Mancini et al. 2020). Our group recently demonstrated that extracellular miRNA profiles are also significantly associated with specific environmental factors, including smoking (Francavilla et al. 2021) or specific dietary habits (Tarallo et al. 2022). Hence, miRNA profiles may represent a promising source of molecular biomarkers for the assessment of the impact of environmental exposure on male germ cells.

The present pilot study aims to evaluate the effects of environmental pollution by testing multiple potential molecular and chemical markers in human semen and germ cells. The  research was conducted on semen from two groups of healthy young men from two areas of different environmental pressures in the Campania Region (Southern Italy). The first one is the Land of Fires (LF), a high environmental impact area that has been plagued by significant pollution issues for decades (Senior and Mazza 2004). The second area is the Valley of the Sele’s Land (VSL), a low environmental impact area situated at ~ 150 km from the LF. We performed canonical spermatozoa analyses (spermiogram), together with a molecular characterization in the same matrix. In detail, we investigated the Protamines/Histones (PRM/H) ratio, the PRM1 and PRM2 levels, the changes in the properties of SNBPs, the seminal trace metal concentration, and simultaneously evaluated miRNA and other sncRNA expression profiles. To the best of our knowledge, this is the first work that correlates seminal miRNA expression with SNBP alterations at the molecular level and contaminants in semen in two areas with different environmental impacts. The results presented in this work are part of the EXPOMAP project (Exposomic mapping of chemical external/internal exposures of communities in high/low pollution areas) within a wider multidisciplinary and multicenter biomonitoring project (https://www.ecofoodfertility.it/).

## Methods

### Recruitment and inclusion criteria

The study was conducted on the semen of healthy young men, homogeneous in age, body-mass index (BMI), and lifestyles, and residing in the LF or the VSL. These two geographical regions were selected based on their opposite environmental pressure. Specifically, the LF area is an open-air dumping ground for Italy’s toxic urban and industrial waste, including heavy metals, sewage sludge, battery acid, asbestos, and radioactive waste. The ecological alarm was raised by a 2011 report by the Regional Environmental Agency (ARPAC), which identified LF among seven contaminated ‘macro-areas’ covering 2.7 million m^2^ with an estimated waste load of 17.4 million m^3^, where studies of soil and groundwater samples had shown high levels of heavy metals (iron, lead, arsenic, manganese). A 2012 Legambiente report, summarizing research into waste trafficking in Campania, suggested that soil and groundwater in several areas of Naples-Caserta were contaminated with inorganic (e.g. fluoride, heavy metals) and organic (e.g. dichloromethane, tetrachloroethylene, toluene) pollutants, forcing local authorities to ban agricultural use of wells in several municipalities in the two provinces (Alberti 2022). Conversely, the VSL is recognized as a region with a low environmental pressure and it is characterized by lower concentration of pollutants and low/medium diffuse emission of sulfur oxide, nitrogen oxide, carbon monoxide, volatile organic compounds, and suspended particles (Pizzolante et al. 2021).

Biological samples, including semen, from the study participants were collected at the Andrology and Lifestyle Medicine Service "Oliveto Citra Hospital" for the Upper Middle Sele Area (ASL Salerno) and at the Villa dei Fiori Clinic in Acerra (Municipalities in the Northern Area of Naples). The inclusion criteria for recruitment were the following: men between the age of 18 and 22 years; healthy; non-regular (daily) alcohol users (≤ 5 alcoholic units per week); not drug users; not previous oncological diseases and/or chemo/radiotherapy; abdominal circumference < 102 cm.

### Sample collection

After 3–4 days of sexual abstinence, semen was collected in the morning by masturbation and stored in sterile containers. In line with the World Health Organization recommendations (World Health Organization 2021), regular tests were performed on sperm samples to assess their quality (volume, pH, sperm concentration, total and progressive motility, morphology, and round cell concentration). After complete liquefaction at 37 °C for 20–30 min, sperm analysis was performed using the phase contrast microscope (Nikon Ci-L) for optical observation with a Makler counting chamber and the Lenshooke Semen X1 Pro system (Bonraybio Co., LTD. Dali Dist., Taiwan), an advanced CE IVD-certified automated analytical system for spermatozoa evaluation.

### SNBP extraction from spermatozoa

Semen was divided into 500 µL aliquots in 1.5 mL tubes. Sperm pellets were obtained by centrifugation for 5 min at 9000 ×*g* at 4 °C. The methodology for the extraction of the SNBP (i.e., histones and protamines) from human semen samples was performed as described by (Soler-Ventura et al. 2018) with few modifications. The detailed protocol applied for the SNBP extraction is reported in the Additional file 1: Methods.

### Electrophoretic analysis of SNBPs

Acetic acid-urea polyacrylamide gel electrophoresis (AU-PAGE) was used to analyze the SNBPs extracted from spermatozoa. AU-PAGE was performed as previously described in (Soler-Ventura et al. 2018), using 15% (w/v) acrylamide (acrylamide: bisacrylamide 30:0.2). After gel polymerization, the procedure described in (Fioretti et al. 2012) was followed. After the electrophoresis, gels were stained with Coomassie Blue Brilliant R-250 as previously described (Vassalli et al. 2015). Protocol details are reported in the Additional file 1: Methods section.

### Plasmid DNA preparation and analysis of SNBP/DNA binding

A pGEM3 plasmid (2867 bp) from *E. coli* HB 101 cells was prepared by using the ZymoPURE™ Plasmid Midiprep Kit (Zymo Research Europe, Germany). For the EMSA and DNA protection assays, the circular form of the plasmid DNA (pDNA) was used. SNBP/DNA binding was analyzed by EMSA as described in (Lettieri et al. 2021). With this assay, the DNA binding ability of SNBP on the base of the SNBP/DNA ratio necessary to obtain DNA saturation (i.e., when DNA is all close to the well) was evaluated. The experiment was carried out on a 1% agarose gel in TBE 1X buffer. Samples were prepared with a fixed amount of pDNA (150 ng) and an increasing amount of SNBPs to obtain samples with different protein/DNA w/w ratios, as previously described (Lettieri et al. 2019). Protocol details are reported in the Additional file 1: Methods section.

### Evaluation of the ability of SNBPs to protect or induce oxidative DNA damage

SNBPs were used to test their ability to protect the pDNA to oxidative damage in pro-oxidative conditions obtained with 10 µM H_2_O_2_ and 5 µM CuCl_2_. The procedure was previously described in (Piscopo 2019). Sample preparation was performed as follows: a fixed quantity of pDNA (150 ng) and an increasing amount of SNBP for obtaining SNBP/DNA ratio (w/w) of 0.4, 0.6, and 0.8, were used. Four categories of DNA damage have been defined: 0, 1, 2, and 3. The level indicated as 0 corresponds to no detectable DNA damage, while the categories from 1 to 3 indicate a progressively high DNA damage level (Additional file 1: Figure S1). Protocol details are reported in the Additional file 1: Methods section.

### Analysis of trace metals in semen samples

Semen samples for the quantification of trace elements were collected in a metal-free vial and frozen for transport to the laboratory. An aliquot of 500 µL was digested with 250 µL of HNO_3_ ≥ 69% (v/v) in a 10 mL quartz vessel using a microwave system (ETHOS™ UP, Milestone Srl, Italy), with a programmed temperature of 15 min for heating up to 160 °C, maintained for 15 min and then 15 min for cooling. Finally, the obtained solution was  brought to a volume of 10 mL with a solution of HNO_3_ ≥ 2% (v/v). Hg, Li, Be, B, Al, V, Cr, Mn, Fe, Co Ni, Cu, As, Se, Sr, Mo, Ag, Cd, Sn, Sb, Ba, Tl, Pb, U, Zn, Ca, K, Na and Mg concentration measurements were performed by Inductively Coupled Plasma Mass Spectrometry (Aurora M90, Bruker, US) and by Microwave Plasma Atomic Emission Spectroscopy (MP-AES 4210, Agilent, US) (Nunzio et al. 2022).

### Total RNA extraction

Total RNA from semen samples was extracted using the Maxwell® RSC miRNA Tissue (Promega) following the manufacturer’s instructions. Initially, 200 µL of sperm samples were centrifuged for 15 min at 12,000 ×*g* at 4 °C. The supernatant was then discarded and 200 µL of phosphate-buffered saline (PBS) was added to the pellet and then centrifuged for 30 min at 5000 ×*g* at 4 °C. At the end of the automated extraction, RNA samples were eluted in 50 µL of water. RNA concentration was measured with a Qubit fluorometer using Qubit microRNA and RNA Broad range (BR) Assay Kits (both from Invitrogen).

### Evaluation of *PRM1* and *PRM2* mRNA levels

*PRM1* and *PRM2* expression levels were measured by RT-qPCR technique performed on the RNA extracted from semen samples of 32 individuals (18 LF and 14 VSL subjects). Protocol details are reported in the Additional file 1: Methods section. The *PRM1* and *PRM2* expression levels were normalized to *ACTB* as a housekeeping gene using the 2−ΔCt and 2−ΔΔCt formulas. Ct values were normalized by subtracting the Ct value of the selected endogenous controls from each of the genes of interest. Differential gene expression was determined by the Wilcoxon Rank-Sum test. p < 0.05 were considered as statistically significant.

### Small RNA-sequencing library preparation

Small RNA-sequencing (small RNA-seq) libraries were prepared using the NEBNext® Multiplex Small RNA Library Prep for Illumina® (New England Biolabs, Inc.) kit as described in (Tarallo et al. 2019). Libraries were pooled together and further purified with a gel size selection. A final Bioanalyzer® 2100 run with the High Sensitivity DNA Kit (Agilent Technologies) allowed the assessment of DNA library quality regarding size, purity, and concentration. The obtained libraries were subjected to the Illumina sequencing pipeline on Illumina NextSeq500 sequencer (75 cycles, Illumina Inc., USA). Protocol details are reported in the Additional file 1: Methods section. The raw reads and the read count table were deposited on Gene Expression Omnibus with the identifier GSE237651.

### Computational and statistical analysis

Statistical analyses on clinical data, spermiogram, SNBP properties, and trace metals were conducted with R (v.4.3.0.). Graph Pad Prism 9 was used to plot the data. R was also employed to compute p-values using the Wilcoxon Rank-Sum test for continuous data and Chi-square for categorical parameters. The correlation analyses were performed using the Spearman's Rank method (cor.test R function).

The small RNA-seq analyses were performed according to (Francavilla et al. 2023) aligning the reads on miRBase v22.1 hairpin sequences with BWA. The miRNA-unmapped reads were aligned on other sncRNAs shorter than 80nt from RNAcentral v22 using BWA. Read count normalization and differential expression analysis were performed with DESeq2 v1.40.1. A miRNA was considered detected if supported by more than 15 normalized reads and as differentially expressed (DE) if associated with a median number of reads greater than 15 in at least one study group and a p < 0.05. Details of the bioinformatic analysis are reported in Additional file 1: Methods.

The analysis of publicly available miRNA expression profiles in seminal samples (datasets GSE110190, GSE36566, and GSE159155) was performed using GEO2R in default settings. For the dataset GSE36566, reporting miRNA profiling of differentiating mice spermatocytes, a differential expression analysis was performed between gonocytes and the other available germ cell population (spermatogonia, round spermatids, and pachytene spermatocytes). The mice homologous miRNA annotations were identified based on their sequence similarity with human DE miRNA sequences reported in miRBase.

## Results

### Characteristics of the study population and spermiogram analysis

**Fig. 1:**
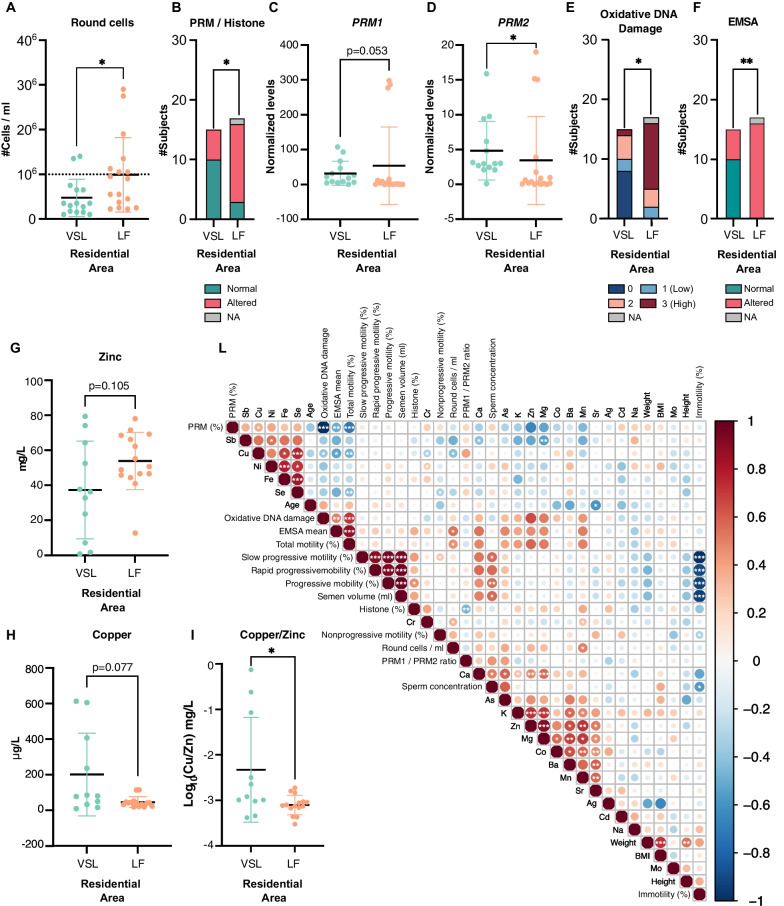
Comparison between spermiogram, SNBP properties, and trace metals in sperm samples of the two study groups. **A** Boxplot of the round cell concentration (round cells/ml). **B** Bar plot of the protamine/histone ratio (PRM/H). **C**, **D** Boxplots of *PRM1* (Panel **C**) and *PRM2* (Panel **D**) expression levels evaluated by RT-qPCR in sperm cells. Results are presented as mean ± SD. **E**. Bar plot of the fraction of samples characterized by a specific category of oxidative DNA damage level (from 0 = no damage to 3 = highest level of damage). **F** Fraction of samples associated with normal or altered electrophoretic mobility shift assay (EMSA) values. **G**, **H** Boxplot of Zinc (**G**) and Copper (**H**) semen levels. Results are presented as mean ± SD. **I** Boxplot of the log_10_ Copper/Zinc ratio. Results are expressed as mean ± SD. p-value from Wilcoxon Rank-Sum test. **L** Correlation plot between spermiogram parameters and levels of metals analyzed in the semen. The dot size and color are proportional to the Spearman correlation (Rho) coefficient. ***p < 0.001, **p < 0.01, *p < 0.05

Demographic information of the 33 subjects included in the study are summarized in Table 1. No significant differences (p ≥ 0.05) in BMI, weight, and smoking habits were observed between VSL and LF subjects (Table 1). Spermiogram analysis did not show any significant differences between samples from the two residential areas in terms of semen characteristics (volume, viscosity, concentration, and sperm motility; Table 1). Globally, over two-thirds of samples in both groups were characterized by semen volumes and concentrations in the healthy range. Conversely, the total and progressive motility were low in 53% and 38% of the subjects, respectively, from VSL and LF. The concentration of round cells (leukocytes/immature germ cells) was significantly higher (p < 0.05) in LF subjects than in those from VSL (Fig. 1A).

**Table 1:** Table summarizing the demographic information and spermiogram analysis of the subjects from the two residential areas (LF and VSL)

Residential area	VSL (n = 15)		LF (n = 18)			
Covariate	N (%)	Mean (SD)	N (%)	Mean (SD)	p-value Wilcoxon Rank-Sum	p-value Chi-square
Age		18 (0.4)		18 (1.0)	0.11	
Weight		77 (11.0)		75 (10.0)	0.83	
BMI		24 (2.6)		24 (2.8)	0.54	
Smoking						
No	14 (93.0)		17 (94.0)			1.00
Yes	1 (7.0)		1 (6.0)			
Sperm concentration (million)		39 (26.0)		48 (40.0)	0.65	
Altered (< 15)	3 (20.0)		6 (38.0)			0.50
Normal (≥ 15)	12 (80.0)		10 (62.0)			
Rapid progressive motility (%)		11 (10.0)		15 (11.0)	0.29	
Slow progressive motility (%)		17 (12.0)		21 (9.9)	0.34	
Nonprogressive motility (%)		14 (9.3)		13 (7.9)	0.95	
Immotility (%)		58 (22.0)		51 (23.0)	0.41	
Progressive motility (%)		28 (21.0)		36 (20.0)	0.29	
Altered (< 32)	8 (53.0)		6 (38)			0.60
Normal (≥ 32)	7 (47.0)		10 (62)			
Total motility (%)		42 (22.0)		49 (23.0)	0.40	
Altered (< 40)	6 (40.0)		4 (25.0)			0.61
Normal (≥ 40)	9 (60.0)		12 (75.0)			
Semen volume (ml)		2.4 (1.2)		2.6 (1.4)	0.74	
Altered (< 1.5)	4 (27.0)		4 (25.0)			1.00
Normal (≥ 1.5)	11 (73.0)		12 (75.0)			
Round cells (10^6^ cells/mL)		0.5 (0.4)		1.0 (0.8)	0.04	
Epithelial cells (%)						
High	3 (20.0)		3 (19.0)			0.98
Intermediate	6 (40.0)		6 (38.0)			
Low	6 (40.0)		7 (44.0)			
Semen viscosity						
V1	12 (80.0)		13 (81.0)			0.72
V2	2 (13.0)		1 (6.0)			
V3	0 (0.0)		1 (6.0)			
V4	1 (7.0)		1 (6.0)

### Analyses of protamine gene expression and protein levels and properties

Since oxidative DNA damage is one of the most impactful causes of male infertility, and protamines are crucial for spermatozoa chromatin compaction and DNA protection from oxidative damage, the protamination rate, expressed as a PRM/H ratio, and the DNA-binding capacity of SNBPs were assessed. A significantly different PRM/H ratio was observed between samples from the two areas (p < 0.05, Fig. 1B**, **Table 2). A canonical ratio (i.e., 85% protamines/15% histones) was found only in approximately 19% of the LF samples compared to 67% of the VSL individuals. In addition, spermatozoa samples of about 6% of LF subjects were characterized only by histones. The altered amount of PRM proteins in LF samples was also confirmed by RT-qPCR analysis, which showed significant decreasing *PRM2* levels in LF samples (p < 0.05, Fig. 1C), and a slight, albeit not significant, increase of *PRM1* levels (Fig. 1D).

**Table 2:** Table summarizing the statistical analyses performed on oxidative DNA damage markers performed on the seminal fluid of subjects from the two residential areas (LF and VSL)

Residential area	VSL		LF			
Variable	N (%)	Mean (SD)	N (%)	Mean (SD)	p-value Wilcoxon	p-value Chi-square
SNBP (%)						
Protamine		73.0 (19.0)		70.0 (15.0)	0.25	
Histone		27.0 (19.0)		30.0 (15.0)	0.25	
Altered protamine/histone ratio						
Yes (≠ 85%/15%)	5 (33.0)		13 (81.0)			0.02
No (= 85%/15%)	10 (67.0)		3 (19.0)			
PRM1/PRM2 ratio		1.2 (0.1)		1.2 (0.2)	0.78	
Altered PRM1/PRM2 ratio						
Yes (< 0.8 or > 1.2)	6 (50.0)		6 (43.0)			1.00
No (range 0.8–1.2)	6 (50.0)		8 (57.0)			
Altered EMSA results						
Yes (> 2.3)	5 (33.0)		16 (100.0)			3.3E-03
No (range 1.6–2.3)	10 (67.0)		0 (0.0)			
Oxidative DNA damage categories*						
Grade 0	8 (53.0)		0 (0.0)			9.1E-03
Grade 1	2 (13.0)		2 (11.8)			
Grade 2	4 (27.0)		3 (17.7)			
Grade 3	1 (7.0)		11 (64.7)			

*Categories from 0 to 3 indicate increasing oxidative damage (see Material and Methods)

A different in vitro capacity of SNBP to protect DNA from oxidative damage was also found between VSL and LF (p < 0.001**; **Fig. 1E). For LF subjects, the majority (70%) of the samples presented high DNA damage (i.e., category 3), while none of them presented no DNA damage (i.e., category 0). Conversely, in VSL samples, only about 20% of the samples were in Category 3, 26% resulted in Category 1 and 2, and more than half of the samples presented no DNA damage (category 0) (Fig. 1E). The four categories of DNA damage are explained in the Additional file 1: Materials (Additional file 1: Figure S1).

The SNBP/DNA binding was evaluated by electrophoretic mobility shift assay (EMSA). The results of the assay showed that all the tested samples from LF subjects presented an altered value of the ratio SNBP/DNA necessary for DNA saturation (p < 0.01; Fig. 1F). In contrast, 33% of the VSL samples showed an altered SNBP/DNA ratio, while for the remaining VSL samples the SNBP/DNA ratio was within the physiological range from 1.6 to 2.3.

### Trace metals detection in semen samples

Since the concentration of trace metals in semen may adversely affect the male reproductive system, the concentration of 29 trace metals was measured in a subset of samples of both groups (Additional file 2: Table S1). Although the total number of metals detected per subject between the two residential areas was not significant, the levels of copper (Cu) (Fig. 1G) and zinc (Zn) (Fig. 1H) were, respectively lower and higher in LF samples. In addition, the Cu/Zn ratio, previously shown to be related to male fertility (Yuyan et al. 2008; Zhao et al. 2016), was significantly lower in LF (p < 0.05, Fig. 1I). More than half of LF samples (n = 8) were characterized by Arsenic (As) levels higher than the detection threshold (0.5 µg/L) while, in VSL, only three out of 11 samples had detectable As levels.

Correlation analysis between trace metal levels, spermiogram parameters, and oxidative DNA damage levels showed two distinct clusters of significant relationships (p < 0.05, Fig. 1L). Specifically, Fe, Ni, Se, Cu, and Sb levels positively correlated with the PRM percentage, while negatively correlated with the extent of DNA damage and the EMSA results. Furthermore, Mn levels positively correlated with round cell concentration, and Ca levels correlated with K and sperm concentration, as well as Mg and Zn. Conversely, the other investigated metals grouped in a cluster poorly correlated with the other parameters.

### MicroRNA profiling in seminal cells by small RNA-Seq

Small RNA-Seq was performed on the semen cellular component of 32 samples from the study cohort (14 VSL and 18 LF). An average of 10.2 ± 6.2 million reads was generated for each sample (Additional file 3: Table S2A and Additional file 1: Figure S2A), and an average of 82.1% of them passed the preprocessing and quality control phase with no significant differences in the outcome between samples from the two groups (Additional file 1: Figure S2B). An average of 6.5 ± 3.3% of reads were aligned on human hairpin miRNA sequences, with an average of 451 ± 205 miRNAs detected per sample (Additional file 3: Table S2A and Additional file 1: Figure S2C). Finally, only one sample (LF25) was excluded from the following analyses since it was associated with a low number of reads (< 1500) and clustered aside in a principal component analysis (Additional file 3: Table S2A and Additional file 1: Figure S2D). Overall, LF subjects were characterized by decreased miRNA levels since 2/3 of the detected miRNAs, including those highly expressed (i.e., miR-148a-3p, miR-99a-5p, miR-10b-5p, miR-21-5p), had lower levels in comparison with those detected in the samples of VSL subjects (Additional file 1: Figure S2E-F and Additional file 3: Table S2B).

**Fig. 2:**
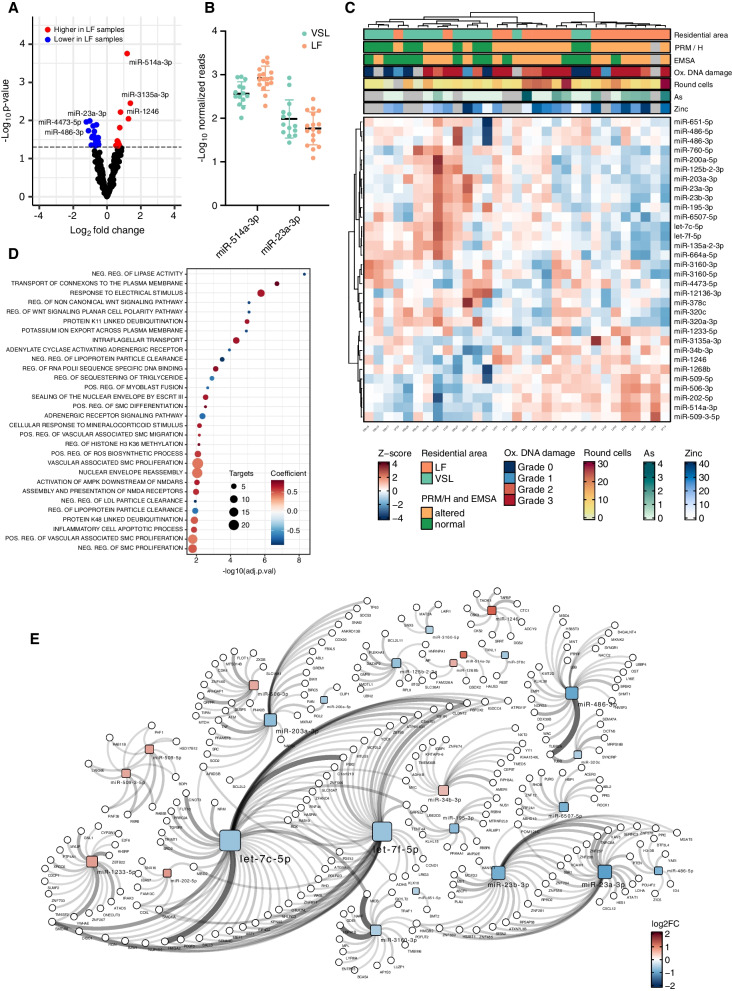
Sperm cell miRNome analyses between the two study groups. **A** Volcano plot of the log2 fold-change (FC) and p-value from the miRNA differential expression analysis. The dashed line indicates the p-value threshold of 0.05. **B** Boxplot for the two top DE miRNAs with opposite expression levels between the two areas. **C** Heatmap of the Z-score-normalized DE miRNA levels. At the top are reported in color code: the residential areas, the PRM/H ratio, EMSA, and oxidative DNA damage classes, the round cell levels, and the Cu/Zn ratio. **D** Dot plot of the most significant processes enriched in the DE miRNA gene targets. The dot size is proportional to the result significance, while the color represents processes predicted to be activated (red) or inhibited (blue) based on the miRNA expression changes. **E** Network of miRNA (squares) and gene target (circles) interactions supported by at least three studies. The node size is proportional to the total number of connections, while the edge thickness is proportional to the number of supporting evidence. Results are expressed as mean ± SD

Differential expression analysis showed 32 DE miRNAs between samples from the two residential areas (p < 0.05) (Fig. 2A and Additional file 3: Table S2B). Specifically, among the 22 miRNAs with decreased levels in LF samples, miR-486-3p, miR-4473-5p, miR-320a-3p, miR-135a-2-3p, and miR-23a-3p showed the most significant differences with respect to VSL samples. Despite the overall decreased miRNA levels in LF samples, 10 out of 32 DE miRNAs had increased levels in comparison with VSL, among which miR-514a-3p, miR-3135a-3p, miR-202-5p, miR-1246, and miR-1233-5p were the most significant. In the same analysis, miR-514a-3p and miR-23a-3p were among the miRNAs with the most significant increasing and decreasing levels in LF samples (Fig. 2B). Clustering analysis based on the DE miRNA levels showed a clear separation between LF and VSL samples (Fig. 2C).

The analysis of validated DE miRNA targets identified 5,771 interactions involving 2,803 genes and 27 miRNAs (Additional file 3: Table S2C). The interactions supported by the highest number of publications were those between let-7c-5p-*HMGA2*, miR-3160-3p-*MICB*, miR-486-3p-*TUBB* (each supported by 16 studies) followed by miR-3160-3p-*HSPA1B* (15 studies) and let-7c-5p-*PBX2* (Auger et al. 2022) (Fig. 2D and Additional file 3: Table S2D). Considering the number of DE miRNAs targeting the same gene, *COPS7B*, *COTL1*, *GPC4*, *LACTB*, *MYLIP*, and *PANK3* were the genes targeted by the highest number of miRNAs (n = 3) whose levels increased in LF samples. In contrast, *ARL6IP1*, *ATXN7L3B*, *NSD1*, *SOD2*, and *TUBB2A* were all targeted by six miRNAs with decreased levels in LF samples (Additional file 3: Table S2E).

Functional analysis of the DE miRNA target genes showed an enrichment of terms (adj. p < 0.05) related to the lipid metabolism (e.g., *NEGATIVE REGULATION OF LIPASE ACTIVITY*, *NEGATIVE REGULATION OF LIPOPROTEIN PARTICLE CLEARANCE*, and *REGULATION OF SEQUESTERING OF TRIGLYCERIDE*), intracellular transport (*INTRAFLAGELLAR TRANSPORT, TRANSPORT OF CONNEXONS TO THE PLASMA MEMBRANE*), response to ROS (*POSITIVE REGULATION OF ROS BIOSYNTHETIC PROCESS*), nuclear envelope and chromatin dynamics (*NUCLEAR ENVELOPE REASSEMBLY,* SEALING *OF THE NUCLEAR ENVELOPE BY ESCRT III*, and *PROTEIN DNA COMPLEX DISASSEMBLY*) (Fig. 2D and Additional file 3: Table S2F).

Network analysis of the miRNA-target interactions showed let-7c-5p and let-7f-5p as the main network hubs, followed by miR-486-3p and miR-23b-3p (Fig. 2E). The levels of all these miRNA hubs decreased in LF samples. Conversely, the network genes targeted by the highest number of miRNAs were *GRPEL2*, *MBD2*, and *SMCR8*, each targeted by three miRNAs.

### Analysis of DE miRNA levels with respect to semen investigated parameters

**Fig. 3:**
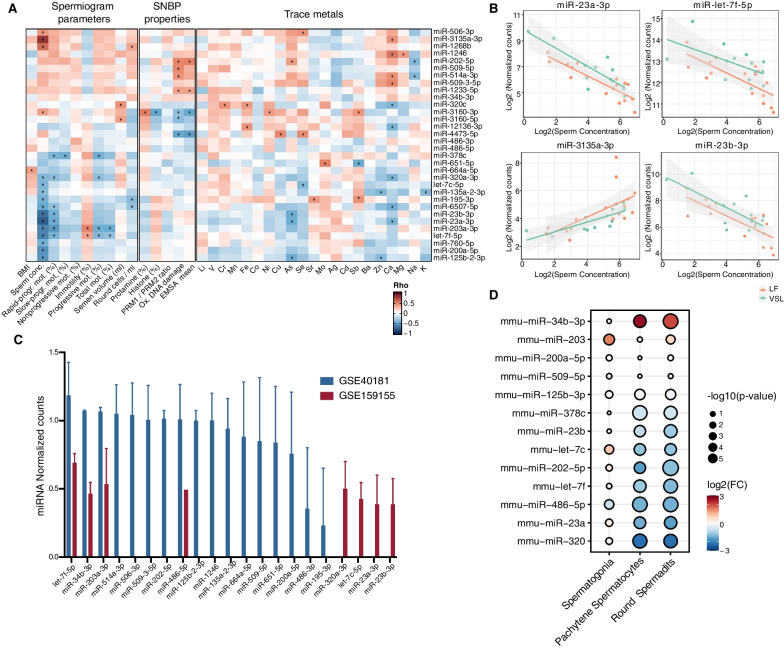
DE miRNA characterization with respect to subject parameters and publicly available miRNA expression profiles. **A** Heatmap of Spearman’s correlation coefficients (Rho) computed between the 32 DE miRNAs levels and the spermiogram parameters, SNBP properties, and trace metals parameters (*p < 0.05). **B** Scatter plots showing the correlations between sperm concentration and miR-23a-3p, miR-3135a-3p, let-7f-5p, and miR-23b-3p expression levels. **C** Bar plot of the normalized expression of the subset of 32 DE miRNAs detected in two publicly available miRNA expression profiles datasets of human semen: GSE40181, (blue) and GSE159155 (red). **D** Comparison between miRNA levels during differentiation of mice germ cells (data from GSE36566): reported miRNAs are the mice homologous of the identified human DE miRNAs in the present study. The log2FCs were computed with respect to miRNA levels in mice gonocytes. The dot color represents miRNA log2FC, while the size is proportional to the p-value of the analysis

A Spearman correlation analysis was performed to investigate the relationships between semen DE miRNA levels and the other investigated parameters (both clinical and biochemical). Twenty-nine out of 32 DE miRNAs were associated with a significant correlation (p < 0.05) with at least one of the analyzed parameters (Fig. 3A and Additional file 4: Table S3). Clustering analysis of the obtained correlations highlighted two main miRNA correlation clusters. The clustering was mainly driven by the correlations with spermiogram parameters, particularly semen concentration (sixteen associated miRNAs) and rapid progressive motility (seven associated miRNAs). Four among the 84 significant correlations, all involving semen concentration, were also confirmed when the analysis was performed on the two study groups separately (Fig. 3B and Additional file 4: Table S3).

### Investigation of DE miRNA levels in publicly available miRNA expression profiles of seminal fluid

The set of identified DE miRNAs was explored in two publicly available datasets reporting miRNA expression profiles data performed on seminal fluids [Reference datasets GSE40181 (Morgan et al. 2020) and GSE159155 (Hua et al. 2019)]. The analysis of semen miRNA profiles from these datasets confirmed the expression of 21 of the 32 DE miRNAs observed in the cellular component of the analyzed samples in the present study (Fig. 3C and Additional file 5: Table S4A). Among them, let-7f-5p, miR-34b-3p, miR-486-5p, and miR-203a-3p were characterized by the highest levels in this biofluid in both analyzed datasets. In addition, a differential expression analysis was performed by stratifying semen miRNA levels based on the in vitro fertilization rate obtained from (Hua et al. 2019). Among the DE miRNAs, let-7c-5p was significantly more abundant in samples with low in vitro fertilization rates, and a similar trend was observed for let-7f-5p, albeit associated with a less significant difference (p = 0.08) (Additional file 5: Table S4B).

Finally, the levels of the 32 DE miRNAs were evaluated in a publicly available miRNA profiling dataset where analyses were performed in cells from different stages of murine spermatogenesis (gonocytes, spermatogonia, round spermatids, pachytene spermatocytes) [Reference dataset GSE36566 (McIver et al. 2012)]. By considering the DE miRNA homologs in mice, the differential expression analysis with respect to gonocyte miRNA levels showed a significant modulation of 10 out of the 32 DE miRNAs (Fig. 3D and Additional file 5: Table S4C). The analysis showed that the mice homologous of eight miRNAs less abundant in LF samples were downregulated in differentiated spermatocytes. Conversely, miR-34b-3p and miR-203, more abundant in LF semen, were upregulated during spermatocyte differentiation (Fig. 3D and Additional file 5: Table S4C).

### Analysis of other sncRNA profiles in seminal cells

**Fig. 4:**
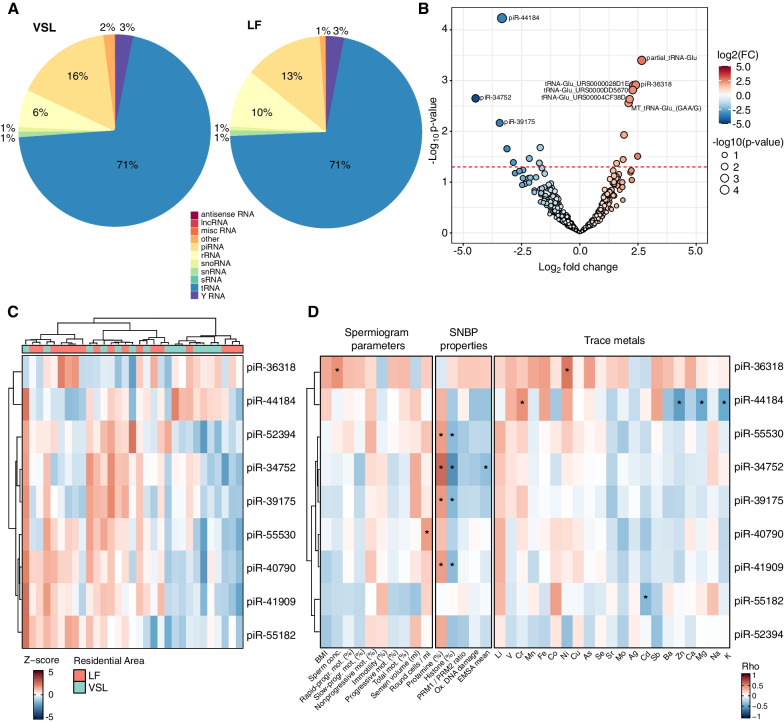
Characterization of other small non-coding RNAs in sperm cells from the two study groups. **A** Pie charts representing the percentage of raw reads mapping on different sncRNA biotypes in the two residential areas. **B** Volcano plot of the log2FC and p-value of the 18 DE sncRNAs between LF and VSL samples. The color of the dots represents the sncRNAs log2FC, while the size is proportional to the p-value of the analysis. Dashed red line indicates the p-value threshold of 0.05. **C** Heatmap of the Z-score-normalized levels of the nine differentially expressed piRNAs in the comparison between LF and VSL samples. **D** Heatmap of Spearman’s correlation coefficients (Rho) computed between the nine differentially expressed piRNAs and spermiogram parameters, SNBP properties, and trace metals parameters (*p < 0.05)

The reads unmapped on any human miRNAs were then explored, to investigate the levels of other sncRNAs. The analysis showed an average of 33.9 ± 0.1% reads aligned to other sncRNAs, particularly piRNAs (14%), tRNAs (71%), and rRNA (8%) (Additional file 3: Table S2A). An average of 1,669 ± 1,017 sncRNAs were detected in each sample (Additional file 3: Table S2A). Samples from the two residential areas did not show major differences in the average percentage of reads assigned to the different sncRNA biotypes, except for the percentage of rRNAs (6% and 10% in LF and VSL, respectively, p < 0.05) and piRNAs (16% and 13% in LF and VSL, respectively, p < 0.1) (Fig. 4A).

Differential expression analysis identified 18 DE sncRNAs between the two groups (p < 0.05): 10 with increasing and 8 with decreasing levels in LF samples (Fig. 4B and Additional file 6: Table S5A). Among the DE sncRNAs, nine (50%) were piRNAs characterized by decreasing levels in LF samples, except for piR-36318 (Fig. 4C). As piRNAs play a relevant role in human spermatogenesis (Wang et al. 2022a), their levels were explored with respect to the other investigated sperm cell parameters (Fig. 4D). Four piRNAs (piR-55530, piR-34752, piR-39175, and piR-41909) were significantly related to oxidative DNA damage. On the other hand, piR-40790 was related to the amount of detected round cells, piR-36318 with semen concentration, and piR-44184 with zinc concentration (Fig. 4D and Additional file 6: Table S5B).

## Discussion

Evidence accumulated over the past decades has demonstrated that declining semen quality may also result from exposure to environmental toxicants, including those from diet/lifestyle (Auger et al. 2022). However, much is unknown about the molecular mechanisms behind these observations, especially the epigenetic alterations by which these factors produce reprotoxic effects. In the present work, alongside conventional analyses, we explored the possible molecular alterations in the semen of young male subjects living in two areas with different environmental pressures. To identify early markers of infertility related to exposure to environmental pollutants, a comprehensive analysis of participants' samples was performed by integrating spermiogram data, oxidative damage, molecular properties of SNBP, the seminal concentration of trace metals, and seminal cell small RNA-Seq data. This analysis showed limited differences between the two residential areas in the alterations in the spermiogram and in the trace metals concentrations. In contrast, alterations in markers of oxidative DNA damage, SNBP molecular characteristics, and miRNA expression profiles were observed.

Interestingly, a strong difference in the protamine/histone ratio (PRM/H) was observed between the two areas. A canonical PRM/H ratio was found in most of the VSL subjects, but only in one-third of LF subjects. In addition, the SNBPs of all LF subjects showed a low DNA binding affinity, while more than half of the VSL samples could protect DNA from oxidative damage. DNA compaction in the sperm head is mediated by the protamines PRM1 and PRM2 and their ratio is critical to sperm fertility. Indeed, an altered PRM1:PRM2 ratio is associated with increased DNA damage, morphological changes, and decreased individual fertility (Barral et al. 2017; Arevalo et al. 2022). Spermatozoa deficient in PRM2 have shown increased expression of PRM1 (Cho et al. 2003). This behavior can be explained as a compensatory mechanism due to the absence of PRM2. In addition, PRM2 seems to play an important role in the morphology of the sperm head (Luke et al. 2016). The observed altered amount of PRM proteins and gene expression, as well as the abnormal PRM/H ratio and SNBPs observed in LF, may be symptoms of an altered fertility, due to the exposure to the pollutants found in the area.

Another important aspect to consider is the histone-to-protamine transition. Indeed, before reaching the almost total protamine composition of sperm DNA, the prevailing SNBPs are histones (Moritz and Hammoud 2022), while in normal mature spermatozoa, protamines represent about 85% of the SNBPs (Torres-Flores and Hernandez-Hernandez 2020). Therefore, histone retention is fundamental for correct genome structuring and epigenetic inheritance, but if this retention is excessive, sperm morphology and subsequent embryonic development may be compromised (Ben Maamar et al. 2018; Ben Maamar et al. 2018). Several factors such as diet, pollutants, and lifestyle can influence the epigenetic pattern of histones and, subsequently, histone retention (Torres-Flores and Hernandez-Hernandez 2020). Thus, we may hypothesize that high pollution in LF may explain the histone alterations observed in the samples from this area.

We did not observe strong differences in trace metals between semen from the two groups analyzed. However, in both areas, we observed some important alterations in the metal levels indicative of decreased fertility. Notably, despite overall LF subjects always had the worst levels of metal traces associated with infertility, in the whole cohort Zn levels were lower than 80–100 mg/L, a level which was previously associated with low fertility (Organization 2021; Colagar et al. 2009; Eggert-Kruse et al. 2002). Zn plays unique roles in males and acts as a hormone balancer for testosterone, prostate, and in sexual functions, as well as an antibacterial agent in the male reproductive system (Allouche-Fitoussi and Breitbart 2020). Zn deficiency hinders spermatogenesis and sperm quality and has a negative effect on serum testosterone concentrations (Hibi et al. 2022). Moreover, in response to increased Zn concentration, Sadli and colleagues observed an enhancement of deacetylation, methylation, and phosphorylation of histone H3 and a decrease in histone H3 lysine 9 acetylation (H3K9ac) (Sadli et al. 2012). H3K9ac is also a key epigenetic modification in the histone/protamine transition (Bao and Bedford 2016) and a possible Zn-induced decrease of H3K9ac is coherent with the high number of LF subjects who showed an altered PRM/H ratio (81%). Protamine PRM2 also differs from PRM1 for Zn binding and physical measurements on intact sperm showed that the PRM2 coordinates one Zn atom per molecule (Bench et al. 2000). Although more analyses are needed, an increase of Zn levels in the LF seminal plasma might be an indirect effect of a decrease in cellular PRM2 levels.

Cu and Se concentrations in semen were lower in LF samples, although the difference between the two groups was not significant. Se and Cu have important antioxidant properties and their deficiency in seminal plasma is associated with increased oxidative stress (Mirnamniha et al. 2019). Moreover, Zn and Cu are important cofactors of superoxide dismutases (SODs), which prevent oxidative stress damage in seminal plasma (Marzec-Wroblewska et al. 2011). In this respect, we found a significantly altered Cu/Zn ratio between the two groups, most likely due to the opposite concentrations of these trace metals. This could cause the reduction of SOD activity, which needs Cu and Zn to reduce oxidative stress in sperm cells. Indeed, a correlation analysis of both groups in this study revealed a negative correlation between Cu and the extent of DNA damage (p < 0.05) and the EMSA results (p < 0.01), confirming the tight binding between these parameters.

Coherently with the literature, we also identified a significant correlation between Zn and Ca levels in the semen samples (Colagar et al. 2009). In fact, in the present study, Ca levels were significantly correlated with Zn, K, Mg, and sperm concentration, which is consistent with the observations reporting a synergistic role of K, Ca, Cu, and Zn in regulating sperm cell hyperactivation in infertile individuals with normal spermiogram (Bolanca et al. 2016). Specifically, the authors reported an anticorrelation between K/Ca and Cu/Zn ratios in the subjects' semen, demonstrating that the increase of K and Zn levels with respect to Ca and Cu, is associated with sperm cell hyperactivation. Similarly, we found a negative correlation (rho = − 0.69) between K/Ca and Cu/Zn ratio, supporting the relevance of considering the synergistic role of trace metals in the characterization of sperm cell fertility.

The correlation between semen Zn levels and male fertility remains a matter of debate, and clinical studies on the role of Zn on male fertility have been inconclusive (Taravati and Tohidi 2016). Low Zn levels in sperm can have a variety of effects on sperm quality and brittleness. It may also affect sperm quality through decreased antioxidant capacity and reduced ability to counteract effects of other heavy metals. The accumulation of toxic heavy metals in the male reproductive system has been proposed to reduce semen Zn concentrations. However, the inhibitory effect of high seminal Zn concentrations on progressive motility was also proposed (Sorensen et al. 1999). Other authors also found a negative correlation between seminal plasma Zn and progressive sperm motility decline. The presence of Zn-binding proteins and ligands in seminal plasma with different Zn-binding capacities, which may alter Zn bioavailability, may be a possible interpretation for this discrepancy. It has been suggested that the presence of unsaturated Zn-binding ligands in semen may have a negative effect on the bioavailability of Zn. Zn in human seminal plasma is distributed among ligands of high, medium, and low molecular weight. Investigation of seminal Zn-binding proteins, combined with Zn levels, will also be relevant in screening asthenospermic subjects, as high unbound Zn may participate in impaired sperm motility in asthenospermia. Negative correlations suggest that despite Zn requirements for normal spermatogenesis and motility, higher levels of this element may be associated with motility deficiencies in asthenozoospermic men. In summary, both low and high levels of Zn in the seminal plasma are detrimental. Adequate Zn levels should therefore be maintained for sperm quality and motility (Taravati and Tohidi 2016).

In addition, albeit not significantly different, the increased levels of As in LF samples may be a direct result of the environmental exposure. The increased exposure to As in recent years can be attributed to various factors, including the widespread use of arsenic-based pesticides in the past and the release of this element from industrial processes (Li et al. 2016). Moreover, As is naturally occurring in soils and rocks, especially in volcanic areas, and the main pathways of human exposure are soil, air, water, and food (Mirza et al. 2014; Esposito et al. 2018). In the LF, one of the significant environmental concerns is the illegal dumping and burning of toxic waste, leading to soil, water, and air contamination, where As is among the multiple toxic substances found, posing significant health risks to the local population (Alberti 2022).

Globally, the analysis of the whole miRNome of the sperm cellular fraction showed profiles consistent with those previously observed for spermatocytes, including the high abundance of miR-148-4p, miR-10a-5p, miR-10b-5p, miR-99a-5p, miR-26a-5p (Salas-Huetos et al. 2020; Nixon et al. 2015). However, in LF samples the levels of detected miRNAs were prevalently lower than in VSL samples, with over 60% of miRNAs associated with decreasing levels. This can reflect a general decrease in the number of transcripts from the pre-meiotic to post-meiotic phases of spermatogenesis (Jan et al. 2017) which might also affect miRNA gene expression. Specifically, thirty-two miRNAs had significantly different expression levels between the two study groups. Functional enrichment analysis of DE miRNA target genes showed an upregulation of processes linked to sperm cell biology, like intraflagellar transport, and processes related to the response to ROS and inflammation. Among the genes involved in these processes, *TUBB2A* and *SOD2* were validated targets of the highest number of miRNAs whose levels decreased in LF samples.

*TUBB2A* codes for the Tubulin Beta 2A Class IIa protein, a component of microtubules that are pivotal intracellular structures involved in sperm cell development, intracellular transport, and motility (Gadadhar et al. 2023). Irradiation with carbon ions has been shown to induce a reduction of tubulin in mouse spermatozoa, resulting in low sperm motility (Li et al. 2014). Similar results were obtained in human studies, in which the expression level of beta-tubulin was lower in asthenospermic individuals than in normospermic individuals (Peknicova et al. 2007; Shen et al. 2013). Conversely, *SOD2* codes for Superoxide Dismutase 2, a key antioxidant protein that converts hydrogen peroxide into diatomic oxygen and plays a critical role in sperm cell biology. In mammals, SOD enzymes are highly expressed in semen and seminal plasma, and their decrease in these specimens has been associated with male infertility (Zelko et al. 2002; Sakamoto and Imai 2017). Reduced SOD activity was correlated with impairments of morphology, low concentration, and low motility in infertility patients. On the other hand, a decreased *SOD* gene expression was associated with an increase in ROS-induced DNA damage causing germ cell apoptosis and a consequent decrease in the number of spermatozoa (Yan et al. 2014). The decrease of miRNAs targeting *SOD2* in LF subjects could result in an increase in the expression levels of this gene in spermatozoa, which is in contrast with the increased oxidative damage observed in sperm of LF subjects. However, given the observed altered levels of the key SOD cofactors, Zn and Cu, in seminal plasma, the enzyme activity might be impaired, and the post-translational control of *SOD2* mediated by miRNAs may be not sufficient. More analyses are needed to clarify the effect of altered targeting miRNA and cofactor levels on this protein activity in relationship with different degrees of environmental pressure.

Among the most significant miRNAs whose levels were decreased in LF samples, let-7c-5p, let-7f-5p, miR-23a-3p, miR-23b-3p, and miR-320a-3p were previously observed as tightly regulated during the spermatogenesis (Salas-Huetos et al. 2020). The let-7 miRNA family members are highly expressed in gonocytes and undifferentiated spermatogonia and downregulated in differentiated spermatocytes (Nixon et al. 2015). The levels of let-7f-5p were reported to be negatively related to the fertility rate in normozoospermic subjects (Salas-Huetos et al. 2016). Coherently, we observed a downregulation of the mouse homolog of let-7f-5p and let-7c-5p in the re-analysis of the spermatogenesis data from (McIver et al. 2012). The downregulation of these miRNAs during spermatogenesis is coherent with our analysis in which we observed a significant anti-correlation with the sperm concentration in both study groups. Two other miRNAs with decreasing levels in LF subjects and significantly anti-correlated with the sperm concentrations were miR-23a-3p and miR-23b-3p. The miR-23 family is highly upregulated in the spermatozoa, seminal plasma-derived exosomes, and testicular tissue of subfertile versus fertile men (Abu-Halima et al. 2019; Abu-Halima et al. 2021). miR-23a-3p and miR-23b-3p share an identical seed sequence and they can target many spermatogenesis-specific genes such as *PFKFB4*, *HMMR*, *SPATA6*, *TEX15*, *SOX6*, and *NOL4* (Becker et al. 2023). These genes have been identified as involved in the spermatogenesis process and their downregulation by an aberrant miRNA increased expression could impair male fertility (Abu-Halima et al. 2019). Finally, the levels of the mouse homolog of miR-320a-3p (mmu-miR-320-3p) were reported to progressively decrease during spermatogenesis and a forced expression of mmu-miR-320-3p in germ cells impair fertility in male mice (Zhang et al. 2018). In Sertoli cells, this miRNA targets *GLUT3* with a consequent reduction of glucose intake and lactate production, a metabolite pivotal for spermatogenesis (Rato et al. 2012).

In contrast, among miRNAs with significantly increased levels in LF samples were miR-34b-3p, miR-202-5p, miR-509, miR-514, and miR-506-3p. The miR-34/449 family, including miR-34a, miR-34b, and miR-34c, not only modulates cell cycle progression, senescence, and apoptosis (Hermeking 2010), but acts as a pivotal regulator of spermatogenesis (Wu et al. 2014; Wang et al. 2022b). The deletion of miR-34b and miR-34c in mice led to a widespread spermatogenesis impairment, associated with low sperm count, a high rate of deformed and not motile sperm cells, and an infertile mouse phenotype, which was also confirmed in humans (Wu et al. 2014; Otto et al. 2017). Indeed, the levels of these miRNAs in human-purified spermatozoa and testicular biopsies are related to the fertility rate (Abu-Halima et al. 2014; Pantos et al. 2021). One study also showed that miR-202-5p targets genes involved in the regulation of spermatogenesis, germ cell development, apoptosis, meiosis, homologous recombination, and other p53-related pathways (Yang et al. 2013). This miRNA is characterized by a testis-specific expression and its levels drastically increased during the differentiation from spermatogonial stem cells to round spermatids (Chen et al. 2017). Knock-out of this miRNA in mice resulted in a precocious spermatogonial differentiation and meiotic initiation driven by upregulation of *DMRT6* gene expression (Chen et al. 2021). Consistently with these observations, other miRNAs increased in LF samples (including miR-509, miR-514, and miR-506-3p) are, in general, downregulated in germinal cells of infertile subjects (Abu-Halima et al. 2014; Sun et al. 2019). These miRNAs belong to a wide cluster of miRNA loci annotated within the lncRNA *LOC105373347* in the Fragile-X region of Chromosome X (ChrXq27.3). miRNAs of this cluster were observed to be transcribed during spermatogenesis and target the *FMR1* gene (Ramaiah et al. 2019).

The evidence from these miRNA profiles in LF samples seems to reflect an increase in altered sperm cell differentiation in these subjects. This is supported by the correlation analysis between miRNA levels and subjects’ covariates. Indeed, upregulated miRNAs in LF were positively correlated with the sperm concentration while those downregulated were negatively related in both LF and VSL subjects. The increasing rate of sperm DNA oxidative damage and round cells observed in LF subjects prompted us to hypothesize a candidate feedforward regulatory loop between a chronic inflammation driven by environmental exposure and a compensatory increase in sperm cell formation. However, additional experimental data are needed to investigate this hypothesis.

We also observed a significant decrease in piRNA levels in LF samples. This is of particular interest given the well-known role of piRNAs in regulating transposable elements and gene expression during spermatogenesis (Wang et al. 2022a). Several pieces of evidence reported a relationship between an altered piRNA activity (e.g., by germline mutations of piRNA-interacting proteins) and human male infertility (Mann et al. 2023). The altered piRNAs presented hereby are described for the first time and further studies will be necessary to elucidate their role in the sperm.

As a pilot study, this work has clear limitations, including a relatively small sample size and a lack of independent validations of the findings. Furthermore, additional in vitro experiments are needed to understand the molecular links between the observed cellular and molecular alterations. Given the lack of additional samples collected from the same subjects, we were not able to investigate other aspects, such as the conformational changes of the protamines that are often induced by environmental pollutants and change their properties, as well as any alteration in the release of protamines from the sperm nuclei, as previously demonstrated by us for *Mytilus galloprovincialis* (Lettieri et al. 2021; Carbone et al. 2023). Further analyses are needed to evaluate whether these pollution-driven alterations in the semen reflect epigenetic alterations in the reproductive tissue, particularly in the epididymis, given its important role in the spermatogenesis. During their passage through the epididymis, spermatozoa acquire the characteristics of motility and fertilization since sperm cells are considered functionally immature when they are released from the testes (Machado-Neves 2022). Other functions attributed to the epididymis include sperm concentration and transport, immune protection of the male gamete, and sperm storage. All these functions can be affected by heavy metals, because of the dysregulation of androgens, the imbalance between pro-oxidants and antioxidants, and the inactivation of proteins because of their reactivity in biological systems. Therefore, epididymis plays a critical role in sperm maturation, and it is a very sensitive organ to heavy metal poisoning. In fact, several studies have reported adverse effects on the epididymis from inorganic As (iAs^+3^/iAs^+5^), cadmium (Cd^+2^), lead (Pb^+2^) and mercury (Hg^+2^/CH_3_Hg^+2^). Exposure to As, Cd, Pb and Hg causes a negative response in epididymal weight, sperm count, sperm motility, hormone levels/production and sperm production. Exposure to metalloids decreases sperm motility, induces oxidative stress through the imbalance of ROS production and scavenging, increases luminal fluid pH (from 6.5 to 7.4) by means of cadmium chloride, decreases sperm viability, and delays sperm transit time via modulation of norepinephrine and dopamine secretion (Machado-Neves 2022). The toxic effect of heavy metals at the hormonal level is probably exerted in two ways. First, the overproduction of free radicals that attack Leydig cells alters morphology, number, and function, leading to cell death. Second, heavy metals can inactivate the enzymes 3 beta-hydroxysteroid dehydrogenase and 17 beta-hydroxysteroid dehydrogenase by forming metal–ligand complexes that interfere with Leydig cell function (Goutam Mukherjee et al. 2022). In addition to the epididymis, albeit to a lesser extent, Pb, Cd, and Zn are accumulated by the seminal vesicles. The latter, in turn, have a greater capacity to accumulate these pollutants than the testis and prostate (Oldereid et al. 1993). The evidence shown in this work adds other important details to understand the reprotoxicity mechanisms of pollutants on germ cell function.

## Conclusions

To the best of our knowledge, this is the first study addressing the molecular mechanisms relating the exposure to environmental pollution and spermatogenesis through the analysis of multiple layers of biochemical and molecular information. The subjects included in the study were carefully recruited to reduce the effect of any possible confounding factors by selecting healthy young male individuals with comparable age, BMI, and lifestyle. Although other validation analyses are needed, the results of this study provide novel data and hypotheses that may help to untangle the complex interaction between environmental exposure and the drastic decrease of male fertility in humans, an effect observed in the general population which is accelerated in men living in polluted areas.

This in-depth identification of the molecular alterations occurring in exposed subjects with comparable spermiograms reinforces the importance of the continuous investigation of precise biomarkers that could indicate early epigenetic alterations impairing the semen quality.

### Supplementary Information

**Additional file 1: **Detailed Materials and Methods and additional file figures. https://doi.org/https://doi.org/10.6084/m9.figshare.24187695.

**Additional file 2: Table S1.** Concentration of trace metals in the analyzed samples. https://doi.org/https://doi.org/10.6084/m9.figshare.24187560.

**Additional file 3: Table S2.** **A **Sample data and small RNA-Seq alignment statistics.** B **Results from the differential expression analysis.** C **List of validated DE miRNA-gene target interactions.** D **Number of studies supporting each miRNA-target interaction.** E **Number of miRNAs targeting the same gene.** F. **Results from the miRNA target enrichment analysis. The log2FCs were coloured using a blue-to-red color scale. https://doi.org/https://doi.org/10.6084/m9.figshare.24187563.

**Additional file 4: Table S3. **Results from the Spearman correlation analysis between DE miRNA levels and analyzed parameters. https://doi.org/https://doi.org/10.6084/m9.figshare.24187566.

**Additional file 5: Table S4.** **A** Levels of DE miRNAs of this study in semen samples from Pauli et al. and Morgan et al. **B** Levels of DE miRNAs of this study in semen samples characterized by high or low fertilization rate. **C** Levels of mice homolog of DE miRNAs of this study in cells from different phases of spermatocyte differentiation. The log2FCs were coloured using a blue-to-red color scale. https://doi.org/https://doi.org/10.6084/m9.figshare.24187548.

**Additional file 6: Table S5.** **A** Results from the differential expression analysis of sncRNA levels between LF and VSL samples. **B** Results from the correlation analysis between the levels of DE piRNAs in our analysis and the semen parameters measured for the study subject. The log2FCs were coloured using a blue-to-red color scale. https://doi.org/https://doi.org/10.6084/m9.figshare.24187551.

## Data Availability

Raw sRNA-Seq data and the miRNA read count tables were deposited on GEO with the identifier GSE237651. Other data analyzed in this study are available in the Additional file Tables or can be requested to the corresponding author upon reasonable request.

## Abbreviations

miRNA: MicroRNA
SNBP: Sperm nuclear basic proteins
EMSA: Electrophoretic mobility shift assay
AU-PAGE: Acetic-urea polyacrylamide gel electrophoresis
piRNA: Piwi-interacting RNA
ROS: Reactive-oxygen species
PRM1: Protamine 1
PRM2: Protamine 2
sncRNAs: Small noncoding RNAs
LF: Land of fires
VSL: Valley of the Sele’s Land
