# Supplementary material for: Small noncoding RNAs and sperm nuclear basic proteins reflect the environmental impact on germ cells
Source: Mol Med. 2024 Jan 20;30:12. doi: 10.1186/s10020-023-00776-6 (PMC10799426; doi:10.1186/s10020-023-00776-6)
Supplement: Supplementary file 1 — Additional file 1: Detailed Materials and Methods and additional file figures. https://doi.org/https://doi.org/10.6084/m9.figshare.24187695. [file 10020_2023_776_MOESM1_ESM.pdf]

Small noncoding RNAs and sperm nuclear basic proteins reflect the environmental impact on germ cells.

Giulio Ferrero, Rosaria Festa, Laura Follia, Gennaro Lettieri, Sonia Tarallo, Tiziana Notari, Antonella Giarra, Carmela Marinaro, Barbara Pardini, Alessandra Marano, Giulia Piaggeschi, Carla Di Battista, Marco Trifuoggi, Marina Piscopo, Luigi Montano, Alessio Naccarati

Supplementary Materials and Methods

SNBP extraction from spermatozoa

The sperm pellet was resuspended with 200 µL of Solution A (20 mM Tris-HCl pH 8, 0.5% Triton X-100, 2 mM $MgCl_2$) and centrifuged for 5 minutes at 8,940x *g* at 4°C. The resulting pellet was resuspended in 200 µL of PMSF 1 mM and centrifuged for 5 minutes at 8,940x *g* at 4°C. The pellet obtained was resuspended in 100 µL of Solution B (20 mM EDTA, 1 mM PMSF, 100 mM Tris-HCl pH 8) and the same volume of Solution C (for 0.5 mL: DTT 5 mM and 6 M guanidinium chloride). Next, the samples were vortexed for 20 seconds, and then 5 volumes of cold (-20°C) 100% ethanol were added to them followed by incubation for 10 minutes at room temperature. The samples were centrifuged at 13,000xg for 15 minutes at 4°C. The supernatant was discarded and 500 µL of 0.5 M HCl at room temperature was added to the pellet and the samples were incubated for 5 minutes at 37°C. Then, samples were vortexed for 20 seconds and thereafter incubated again for 2 minutes at 37°C. The samples were subsequently centrifuged at 17,530x *g* for 10 minutes at 4°C, and 125 µL of 100% trichloroacetic acid (TCA) was added to the supernatant for protein precipitation at -20°C. Samples were centrifuged at 17,530x *g* for 10 minutes at 4°C-80°C. After discarding the supernatant, 500 µL of 1% β-mercaptoethanol in acetone was added and mixed to the pellet, and the samples were then centrifuged at 17,530x *g* for 5 minutes at 4°C. This last step was repeated twice. Finally, the protein pellets obtained were dried in a Savant vacuum desiccator and then resuspended in 55 µL of Milli-Q water for further analyses.

Electrophoretic analysis of SNBPs

Acetic acid-urea polyacrylamide gel electrophoresis (AU-PAGE) was used to analyze the SNBPs extracted from spermatozoa. AU-PAGE was performed using 15% (w/v) acrylamide (acrylamide: bisacrylamide 30:0.2). The gel, with a final volume of 8 mL, consisted of 15:0.1 acrylamide/bisacrylamide, 2.5 M urea, 0.9 M acetic acid, 100 µL TEMED, and 140 µL of 10% APS. After gel polymerization, a pre-run of approximately 1.30 hours was performed at a constant voltage of 150V by using 0.9 N acid acetic buffer as a running buffer. Before the running, 4 µg of proteins were denatured in 20 µL of a solution consisting of 12.8 M β-mercaptoethanol and 8 M urea for 1 hour. After this step, samples were loaded on gel with 2 µL of 100% acetic acid and 2 µL of 0.001% pyronin as a running marker. The electrophoresis was performed at 120 V for about 1.30 hours. The image was acquired using the GelDoc Biorad system (BioRad, Hercules, CA, USA) with Imaging Lab 6.0.1 software (build 34; BioRad, Hercules, CA, USA).

Plasmid DNA preparation and analysis of SNBP/DNA binding

A pGEM3 plasmid (2867 bp) from *E. coli* HB 101 cells was prepared by using the ZymoPURE™ Plasmid Midiprep Kit (Zymo Research Europe, Freiburg, Germany). After extraction of plasmid DNA (pDNA), its concentration was evaluated by spectrophotometer Nanodrop 1000 (Thermo Fisher Scientific, Waltham, MA, USA), while integrity and topological state of plasmid DNA were verified by gel electrophoresis on 1% agarose gel in 89 mM Tris-HCl pH 8.0, 2 mM EDTA, and 89 mM Boric Acid (TBE). For the EMSA and DNA protection assays, the circular form of the plasmid DNA was used. EMSA was carried out on a 1% agarose gel in TBE 1X buffer. Samples were prepared with a fixed amount of pDNA (150 ng) and an increasing amount of SNBPs to obtain samples with different protein/DNA w/w ratios. The pDNA and proteins interacted for 5 minutes at room temperature before adding TBE 1X and dye for running. The electrophoresis was performed at a constant voltage of 100 V for 30 minutes and DNA was visualized using SafeView (Abm, Applied Biological Materials Inc, Viking Way, Richmond, Canada). Gels were acquired with a GelDoc system (BioRad, Hercules, CA, USA) with Imaging Lab 6.0.1 software (build 34; BioRad, Hercules, CA, USA).

Evaluation of the ability of SNBPs to protect or induce oxidative DNA damage

Sample preparation was performed as follows: a fixed quantity of pDNA (150 ng) and an increasing amount of SNBP for obtaining SNBP/DNA ratio (w/w) of 0.4, 0.6, and 0.8, were used. After 5 minutes of interaction between DNA and SNBP at room temperature, H2O2, and CuCl2 were added, and samples were incubated in the dark for 30 minutes in a thermoblock set at 37°C. To avoid EDTA coordination of subsequent metals, Sample Buffer 10X (1X final concentration in the samples) was added right before electrophoresis analysis at incubation end. The samples were electrophoretically analyzed on 1% agarose gel at 100 V for 30 minutes in TBE 1X. The DNA was visualized using SafeView (abm, Applied Biological Materials Inc, Viking Way, Richmond, Canada). Gels were acquired with a GelDoc system (BioRad, Hercules, CA, USA) with Imaging Lab 6.0.1 software (build 34; BioRad, Hercules, CA, USA).

Evaluation of *PRM1* and *PRM2* mRNA levels

Reverse transcription was performed using the Superscript VILO cDNA Synthesis Kit (ThermoFisher) with 200 ng of RNA as starting material and according to the manufacturer's instructions. For qPCR, undiluted complement DNA (cDNA) was mixed with TaqMan Fast Advanced Master Mix and specific TaqMan probes (20x) (ThermoFisher; Assay names: *PRM1*, Hs00358158_g1; *PRM2*, Hs04400446_g1; *ACTB*, Hs01060665_g1) according to the manufacturer's

protocol. All cDNA products were prepared in triplicate/duplicate PCR reactions following the manufacturer's instructions. For quality control purposes, one RNA sample was measured twice and a sample containing nuclease-free water and carrier RNA was profiled as negative control. One RNA sample extracted from the human brain (Invitrogen AM7962) was used as positive control for *ACTB* and negative control for *PRM1* and *PRM2*. All the reactions were run on ABI Prism 7900 Sequence Detection System (Applied Biosystems).

Small RNA-sequencing library preparation

Small RNA-sequencing (small RNA-seq) libraries were prepared using the NEBNext® Multiplex Small RNA Library Prep for Illumina® (New England Biolabs, Inc.) kit used to convert small RNA transcripts into barcoded cDNA libraries. For each library, 200 ng of RNA was processed as starting material. Each library was prepared with a unique indexed primer. Multiplex adapter ligations, reverse transcription primer hybridization, reverse transcription reaction and PCR amplification were performed according to the manufacturer's protocol. After PCR amplification, the cDNA constructs were purified with the QIAQuick® PCR Purification Kit (QIAGEN), following the modifications suggested by the NEBNext® Multiplex Small RNA Library Prep for Illumina® protocol. Final libraries were loaded on a Bioanalyzer® 2100 (Agilent Technologies) using the DNA High Sensitivity Kit (Agilent Technologies) according to the manufacturer's protocol.

Computational and statistical Analysis

Small RNA-Seq eads were quality-controlled and trimmed of adapter sequences using Cutadapt v.3.7. Reads shorter than 14 nt were removed. Surviving reads were aligned on human miRBase v22.1 hairpin sequences using BWA v0.7.12. The pipeline quantifies mature miRNA sequences annotated in miRBase as well as "novel" mature miRNAs identified based on the read mapping position within the hairpin sequence. Annotations with identical sequences were merged into a single annotation. For piRNAs, annotations belonging to the same cluster from piRNAclusterDB were joined into a single piRNA cluster annotation. Functional enrichment analysis was performed using RBiomirGS v0.2.12 considering only validated miRNA-targets interactions from miRTarBase v7.0 and miRecords. The analysis was performed with respect to MSigDB gene sets (version: msigdb_v2022.1.Hs). Only terms associated with an adj. $p<0.05$ and at least three gene targets were considered significant. The miRNA-target interaction network was performed by considering the interactions supported by at least three independent studies and it was graphically represented using Cytoscape v3.9.1.

Supplementary Figures

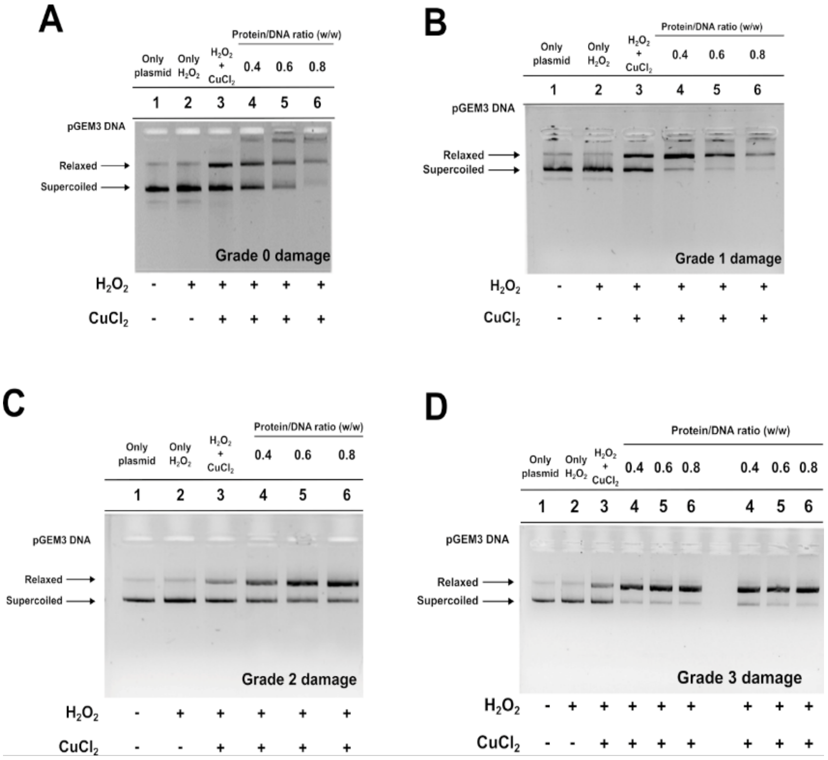

Supplementary Figure 1. Analysis on a 1% agarose gel of the ability of SNBPs to protect DNA damage of the pGEM3 plasmid in the presence of H_2O_2 and $CuCl_2$. The different damage groups are Grade 0 (**A**), Grade 1 (**B**), Grade 2 (**C**) and Grade 3 (**D**). The damage condition purposely created for plasmid DNA by adding 10 µM H2O2 and 5 µM CuCl2 is shown in well 3 of all gels. The addition of SNBPs in the ratios 0.4 to 0.8 to DNA produces: no increase in damage, i.e., no increase in the intensity of the relaxed plasmid DNA band compared to the condition shown in well 3 (Grade 0 (**A**)), a slight increase in the intensity of the relaxed DNA band, but only in the 0.4 SNBP/DNA ratio and a decrease in the 0.6 and 0.8 ratios, compared to the condition shown in well 3 (Grade 1 (**B**)), an increase in the intensity of the band corresponding to relaxed DNA and a high fraction of supercoiled DNA remains, indicating that the SNBPs of these subjects have a low capacity to aggregate DNA (Grade 2 (C)), that almost all the DNA becomes relaxed, suggesting that SNBPs from these individuals have a very low capacity to aggregate DNA and consequently a poor ability to protect DNA from oxidative damage (Grade 3(**D**)).

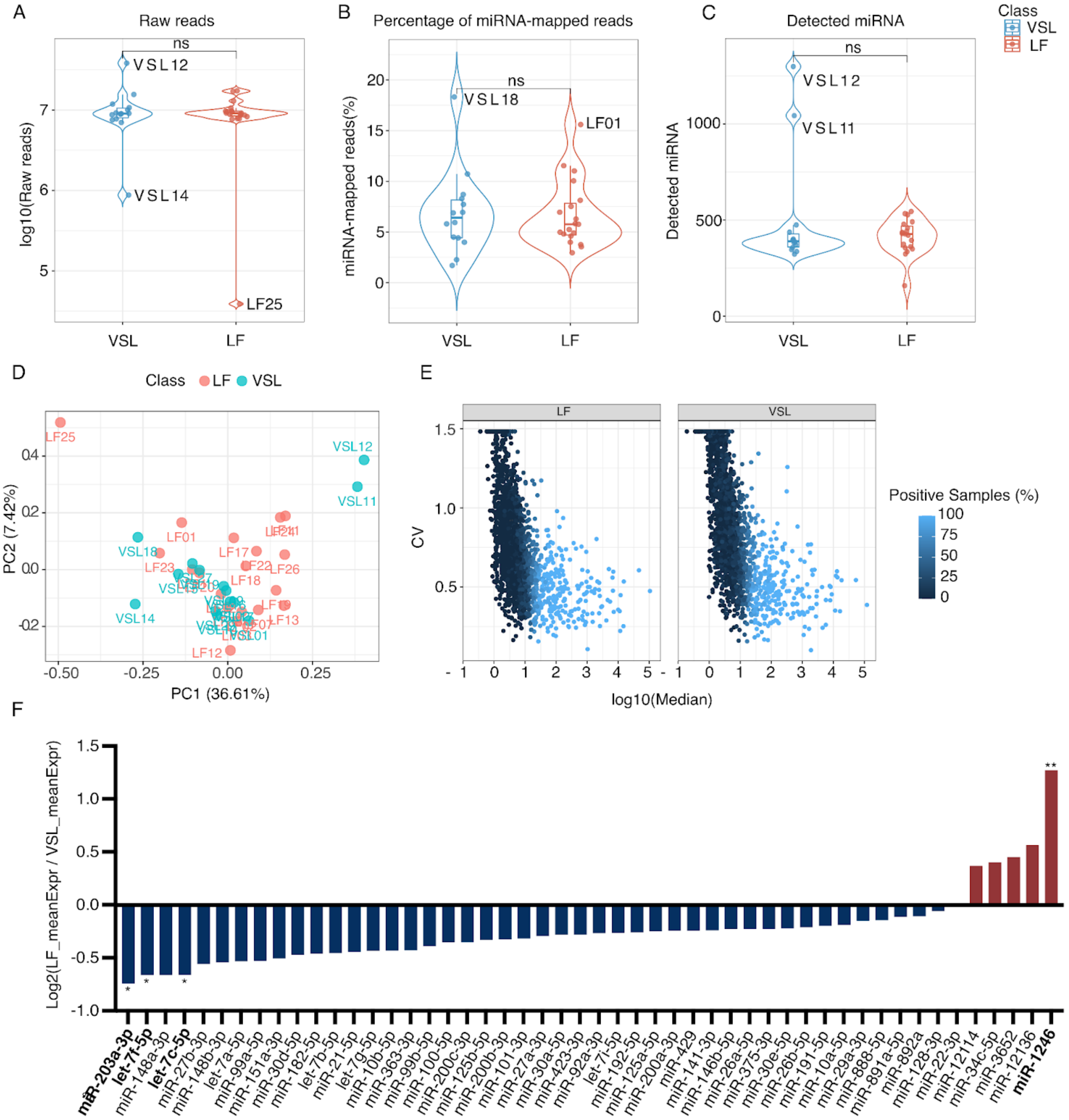

Supplementary Figure 2 A-C. Boxplots of the number of raw small RNA-Seq reads (**A**), the percentage of miRNA-mapped reads (**B**), and the number of detected miRNAs in each sample (**C**). **D.** Principal Component Analysis of the analyzed samples based on the levels of the semen miRNAs. **E.** Scatter plot of the relationship between semen miRNA median levels (x-axis) and the Coefficient of Variance (CV) computed as the ratio between the Median Absolute Deviation (MAD) and the median. The color-code represents the percentage of samples in which each miRNA was detected. **F.** Barplot of the log2 ratio of the mean miRNA levels in LF and VSL samples considering the top 50 expressed miRNAs. The red and blue colors of the bars represent, respectively, miRNA with higher and lower levels in LF with respect to VSL samples. The DE miRNAs in our analysis are reported in bold. **p<0.01; *p<0.05. P-value from DESeq2 analysis.

Supplementary Tables

Supplementary Table 1 (https://doi.org/10.6084/m9.figshare.24187560)

Concentration of trace metals in the analyzed samples.

Supplementary Table 2 (https://doi.org/10.6084/m9.figshare.24187563)

A. Sample data and small RNA-Seq alignment statistics. **B.** Results from the differential expression analysis. **C.** List of validated DE miRNA-gene target interactions. **D.** Number of studies supporting each miRNA-target interaction. **E.** Number of miRNAs targeting the same gene. **F.** Results from the miRNA target enrichment analysis.

Supplementary Table 3 (https://doi.org/10.6084/m9.figshare.24187566)

Results from the Spearman correlation analysis between DE miRNA levels and analyzed parameters.

Supplementary Table 4 (https://doi.org/10.6084/m9.figshare.24187548)

A. Levels of DE miRNAs of this study in semen samples from Pauli et al. and Morgan et al. **B**. Levels of DE miRNAs of this study in semen samples characterized by high or low fertilization rate. **C**. Levels of mice homolog of DE miRNAs of this study in cells from different phases of spermatocyte differentiation.

Supplementary Table 5 (https://doi.org/10.6084/m9.figshare.24187551)

A. Results from the differential expression analysis of sncRNA levels between LF and VSL samples. **B.** Results from the correlation analysis between the levels of DE piRNAs in our analysis and the semen parameters measured for the study subject.
